# Key attributes of patient centered medical homes associated with patient activation of diabetes patients

**DOI:** 10.1186/s12875-017-0704-3

**Published:** 2018-01-05

**Authors:** Lori A. Bilello, Allyson Hall, Jeffrey Harman, Christopher Scuderi, Nipa Shah, Jon C. Mills, Shenae Samuels

**Affiliations:** 1 Center for Health Equity and Quality Research, College of Medicine, UF Health Jacksonville, 6th Floor, Tower II, Suite 6015, 580 West 8th Street, T60, Jacksonville, FL 32209 USA; 2 Department of Health Services Administration, School of Health Professions, University of Alabama at Birmingham, 1720 2nd Ave S, Birmingham, AL USA; 3 Department of Behavioral Sciences and Social Medicine, College of Medicine, Florida State University, 1115 West Call Street, Tallahassee, FL 32306 USA; 4 Department of Community Health and Family Medicine, UF Health Family Medicine and Pediatrics – New Berlin, 3122 New Berlin Road, Jacksonville, FL 32226 USA; 5 Department of Community Health and Family Medicine, College of Medicine, UF Health Jacksonville, 3rd Floor, LRC, 653-1 West 8th Street, C257, Jacksonville, FL 32209 USA; 6 Department of Epidemiology, College of Medicine, University of North Carolina – Chapel Hill, 2103B, 135 Dauer Drive, Chapel Hill, NC 27599 USA; 7 Department of Health Services Research, Management & Policy, College of Public Health and Health Professions, University of Florida, PO Box 100195, Gainesville, FL 32610 USA

## Abstract

**Background:**

Approximately 24 million Americans are living with diabetes. Patient activation among individuals with diabetes is critical to successful diabetes management. The Patient Centered Medical Home (PCMH) model holds promise for increasing patient activation in managing their health. However, what is not well understood is the extent to which individual components of the PCMH model, such as the quality of physician-patient interactions and organizational features of care, contribute to patient activation. This study’s objective is to determine the relative importance of the PCMH constructs or domains to patient activation among individuals living with diabetes.

**Methods:**

This study is a cross-sectional analysis of 1253 primary care patients surveyed with type II diabetes. The dependent variable, patient activation, was assessed using the Patient Activation Measure (PAM). Independent variables included 7 PCMH domains- organizational access, integration of care, comprehensive knowledge, office staff helpfulness, communication, interpersonal treatment and trust. Ordered logistic regression was performed to determine whether each PCMH domain was independently associated with patient activation, followed by a final ordered logistic regression that included all the PCMH domains in a single adjusted model.

**Results:**

Using the full adjusted model, the odds of patients reporting higher activation scores (PAM) were found to be significant in the domains that represented organizational access (OR 1.56, 95% CI 1.31–1.85) and comprehensive knowledge (OR 1.44, 95% CI 1.13–1.85).

**Conclusions:**

Many practices have struggled with the challenge to develop fully functional patient-centered medical homes. In an effort to become more patient-centered, this study aimed to address what factors activated diabetic patients to adhere to diabetes management plan. Understanding these factors can help identify PCMH attributes that practices can prioritize and improve upon to assist their patients in improving health outcomes.

**Trial registration:**

Study was not a clinical trial; therefore it was not registered.

## Background

The Medical Home has emerged as a major organizing construct in the delivery of primary care, having first been recognized by the American Academy of Pediatrics (AAP) in 1967. As the concept has been more fully explored, researched and refined over the last half century, more clearly defined principles and practices have been adopted by key primary care professional societies. It wasn’t fully recognized by the medical establishment until 2007 when the AAP, American College of Physicians, American Academy of Family Physicians, and the American Osteopathic Society published the Joint Principles for a Patient Centered Medical Home (PCMH). In 2008, the National Committee on Quality Assurance (NCQA) adopted criteria for practice recognition as a PCMH. There are 3 levels of NCQA recognition ranging from level 1 to level 3 and require medical practices to demonstrate that they have met certain criteria for the following standards of care: patient centered access, team based care, population health management, care management and support, care coordination and care transitions, performance measurement and quality improvement [1].

Recent studies have provided evidence that PCMHs improve the quality of care, lead to better patient outcomes and experiences with the care process, and reduce emergency department visits and hospitalizations [2–4]. The PCMH model is especially relevant to the care of patients with chronic diseases such as diabetes where it requires close monitoring by the provider, patients involved in the management of their disease, a team-based approach in the continuum of care and adopts many of the components of Wagner’s Chronic Care Model [5, 6]. Several of the Centers for Medicare and Medicaid Services’ (CMS) funded PCMH demonstration projects have reported improvements in Hemoglobin A1c (HbA1c) control and provide encouraging results to support the PCMH model as a viable mechanism to improve the quality of diabetes care [7].

Patient centered care not only recognizes the patient as central to care provision, but it also requires the patient to be involved in their care and to have the knowledge, skills, and motivation to do so. Promoting patient activation is a key principle in a PCMH setting and is essential to the provision of high quality care and to achieve better patient outcomes. Patient activation is the ability of patients to take a pro-active role in managing their health and have the skills, knowledge, and confidence to do so [8] while patient engagement or motivation are often described as the preliminary steps to patient activation where the patient is involved in learning about their condition and making decisions about their health but may not have taken a pro-active role in managing their health. Research has found that engaged, informed, confident, and skilled patients are more likely to perform activities that will maintain or promote their own health [9, 10]. A study by Remmers et al. showed that patients with diabetes who are more activated in their care had better HbA1c control and conformed to the diabetes guidelines in testing for HbA1c and LDL Cholesterol [11].

For practices that are adopting the PCMH model, an important consideration is whether certain constructs or domains of the model have a higher likelihood of activating patients relative to other domains. As described in the Engagement Behavior Framework by the Center for Advancing Health, several components related to the patients’ clinical care, such as seeking appropriate, high quality care, communication with health professionals and organizational access, are important factors for patient engagement [12]. Understanding the relative importance of these medical home domains to patients can guide practices that are converting to the PCMH model develop specific strategies to improve patient activation. The goal of this study is to determine the relative importance of certain well-established PCMH constructs or domains and their association to patient activation among individuals living with diabetes.

## Methods

Patients with diabetes at 4 large family medicine centers were surveyed about their experiences with care and if they took an active role in their health and healthcare. These primary care practices have achieved the highest level of NCQA recognition (Level 3) as a PCMH, are affiliated with an U.S. academic medical center, and participate in a practice based research network in Florida. One practice was located at the academic medical center while the other three practices were located in various sections of a large metropolitan city and had diverse patient populations. There were over 5300 diabetic patients being managed by these 4 practices. Patients who met the inclusion criteria of being 18 years and older with an ICD-9 (International Classification of Diseases–9th Revision) code indicating type II diabetes (codes 250–250.9) who had at least 2 visits within the past 2 years (2012–2013), and who did not opt out were randomly selected to participate in a telephone survey administered by a university based Survey Research Laboratory in 2014. The survey took approximately 10 min for respondents to complete. A power analysis resulted in needing 1301 participants in order to detect a five point difference in domain ratings across the four clinics with 80% power. Sampling occurred until 1301 surveys were completed. The actual analytic sample was reduced to 1253 after observations with missing data were excluded with minimal effect on the power. The response rate for each clinic (the number of complete interviews divided by the number of telephone numbers contacted) ranged from 65 to 73%.

The survey instrument included the Patient Activation Measure (PAM) and items from the Ambulatory Care Experiences Survey (ACES). The PAM is widely used to assess the knowledge, skills and confidence of individuals in managing their own health and their healthcare and has been tested to be a reliable and valid tool for patient activation [8, 13]. Numerous studies have used the PAM tool to assess patient activation in different patient populations such as rural populations, patients with chronic conditions such as diabetes, multiple sclerosis or mental health conditions that have shown that patient activation is positively related to patient self –management [14–18]. Additionally the tool has been translated and validated in other languages included Dutch, German and Danish [19–21].

The ACES instrument includes 11 summary measures of patients’ experiences across 2 domains, quality of physician-patient interactions and organizational features of care, and has been extensively tested, validated and used in numerous studies [22–24]. Using items from seven of the 11 available measures from ACES, we constructed a shortened version of the survey with the following medical home domains: organizational access, comprehensive knowledge, integration of care, communication, office staff helpfulness, interpersonal treatment, and trust. Items selected from the ACES for each domain are listed in Table 1. The domains that were selected best fit the organizational structure of the PCMHs included in the study. The PCMH domains of organizational access, communication, integration of care and comprehensive knowledge are composite measures while office staff helpfulness, interpersonal treatment and trust are single item measures. The composites were developed based on scoring instructions provided by the ACES developers. The instructions provided by the developers pertained to a more comprehensive version of the ACES survey called the Primary Care Assessment Survey. The composite measures were tested for internal consistency and resulted in the following Cronbachs α scores: organizational access (0.86), comprehensive knowledge (0.76), integration and care coordination (0.65), and communication (0.86). Composite measures reflected the mean of the non-missing responses from each item. If the respondent did not provide at least one response to an item within a domain then the observation was considered missing. We dichotomized all composite scores into binary variables to simplify interpretation of the results. We established the threshold of 4.5 based on distributional analysis of the original scores and conducted multiple sensitivity analyses to determine the impact of various thresholds on the outcomes. Sensitivity analysis produced similar results across different thresholds. Observations with a score of 4.5 (mean score for composites) or greater were coded as 1 (a high perception of that PCMH domain) and those below were coded as a 0 (a low perception of that PCMH domain).

**Table 1:** Survey questions from the Ambulatory Care Experiences Survey grouped by PCMH Domains

PCMH domains	Survey questions
Organizational Access	When you needed care for an illness or injury how often did your personal doctor’s office provide care as soon as you needed it?
	When you scheduled an appointment for a check-up or routine care how often did you get the appointment as soon as you needed it?
	When you called your personal doctor’s office with a medical question during regular office hours how often did you get an answer the same day?
	When you called your personal doctor’s office after regular office hours, how often did you get the help or advice you needed?
Integration of Care	When your personal doctor sent you for a blood test, x-ray, or other tests, did someone from your doctor’s office follow-up to give you the test results?
	How often did your personal doctor seem informed and up to date about the care your received from your specialist doctor?
Comprehensive Knowledge	How would you rate your doctor’s knowledge of your medical history?
	In the last 12 months, how often did your doctor seem to know all the important information about your medical history?
Office Staff Helpfulness	In the last 12 months, how often were the office staff at your personal doctor’s office HELPFUL as you thought they should be?
Communication	How often did your personal doctor listen carefully to you?
	How often did your personal doctor give you clear instructions about what to do to take care of the health problems and symptoms that were bothering you?
	How often did your personal doctor explain things in a way that was easy to understand?
Interpersonal Treatment	How often did your personal doctor spend enough time with you?
Trust	How often did you feel you could tell your personal doctor anything, even things you might not tell anyone else?

Adapted from Safran DG et al. [22]

*PCMH* Patient Centered Medical Home

### Analytic variables

The Patient Activation Measure (PAM) was used to assess the level of patient activation. PAM scores were on a 0 to 100 scale and converted into an ordinal categorical variable with four levels based on the PAM developers’ recommendations: 0 to 47; 47.1 to 55; 55.1 to 67; and over 67.

Covariates/socio-demographic variables were collected from questions in the ACES survey and included age; race (minority, non-minority); gender (female, male); health status (low, medium, high); education (less than high school, high school grad or higher); type of insurance (uninsured, Medicare, private, unknown); marital status (not married, married), and PCMH where the individual was a patient.

Data preparation was performed using Statistical Analysis Software (SAS), Version 9.3 and because the PAM scores were divided into 4 ordered categories, ordered logistic regression analyses was performed using STATA SE, Version 13.1. We first modeled each PCMH domain separately (adjusted for covariates). This was done in order to determine whether each PCMH domain was independently associated with patient activation. Then we performed one final ordered logistic regression that included all the PCMH domains in a single adjusted model using the ordered logit procedure in STATA with PAM (4 ordered categories) as the dependent variable. Since patients were sampled within physician practices, we used random effects to account for correlation of patients due to clinical practice characteristics.

## Results

The study sample, which includes patients with diabetes at the 4 PCMHs participating in the study, is representative of an urban population. The majority of participants consider themselves to be a racial or ethnic minority, with only 37.4% identifying as non-Hispanic white. The average age was 60.9 ± 11.61 with a range of 19 to 89 years. Other demographic characteristics include: 74.1% reported having a high school education/GED or higher, 41.6% were married, and 10.6% considered themselves to have low health status. Medicare was the most common insurance coverage (44.8%) followed by Medicaid/uninsured (28.2%) and private insurance (27.0%). Many of the patients have been receiving care from the same medical practice for 3 years or more (62.9%). See Table 2 for details of the study sample characteristics.

**Table 2:** Sample Characteristics (*n* = 1253)

	Percent
Length of time with Provider	
Less than 6 months	8.16
Between 6 months – less than 1 year	7.76
1 year to less than 3 years	21.21
3 years but less than 5 years	16.14
5 years or more	46.73
Gender	
Male	35.51
Female	64.49
Education	
High School diploma or more	74.14
No High School diploma	25.86
Health Status	
Excellent/Very Good	19.06
Good	35.57
Fair/Poor	45.37
Race/Ethnicity	
White non-Hispanic	37.43
Minority	62.57
Marital Status	
Not married	58.42
Married	41.58
Insurance	
Medicaid/Uninsured	28.25
Medicare	44.79
Private	26.96
Mean age in years (SD) 61.93	(11.61)

Table 3 shows the results from the ordered logit models showing the relationship between each PCMH domain individually and PAM scores. The odds of patients reporting higher activation scores (PAM) were found to be significant for each of the PCMH domains: organizational access (OR 2.35, 95% CI 1.89–2.92), integration of care (OR 2.09, 95% CI 1.91–2.28), comprehensive knowledge (OR 2.90, 95% CI 1.07–4.04), office staff helpfulness (OR 2.50, 95% CI 1.70–3.67), communications (OR 3.27, 95% CI 2.94–3.63), interpersonal treatment (OR 2.32, 95% CI 1.61–3.34) and trust (OR 2.67, 95% CI 2.09–3.65).

**Table 3:** Relationship between each individual PCMH domain and likelihood of higher activation score

PCMH domain	Odds ratio	95% Confidence interval		*p*-value
		Lower bound	Upper bound	
Organizational Access	2.54	2.02	3.21	.000
Integration of Care	2.26	1.89	2.69	.000
Comprehensive Knowledge	2.91	2.10	4.04	.000
Office Staff Helpfulness	2.70	1.76	4.15	.000
Communication	3.21	2.63	3.92	.000
Interpersonal Treatment	2.48	1.52	4.03	.000
Trust	2.68	2.02	3.56	.000

Adjusted for: insurance status, age, gender, self-reported health status, racial/ethnic minority status, marital status, length of time with primary care provider (Ordered logit, *n* = 1253)

Table 4 shows the results from the ordered logit model that included all PCMH domains and covariates in one model. The odds of patients reporting higher activation scores (PAM) were found to be significant for only two of the PCMH domains: organizational access (OR 1.56, 95% CI 1.31–1.85) and comprehensive knowledge (OR 1.44, 95% CI 1.13–1.85). Patients in excellent health or good health were more likely to have higher activation scores compared to those who self-rated their health as fair or poor. In addition, our analysis found that individuals who were high-school graduates and were white (non-Hispanic) had higher odds of having a higher PAM score, compared to individuals who were not high-school graduates. Also, as age increases the likelihood of having a higher activation score declined.

**Table 4:** Predictors of higher activation score (Ordered logit, *n* = 1253)

	Odds ratio	95% Confidence interval		*p*-value
		Lower bound	Upper bound	
PCMH domains				
Organizational Access	1.56	1.31	1.85	.000
Integration of Care	1.14	0.93	1.41	.197
Comprehensive Knowledge	1.44	1.13	1.83	.003
Office Staff Helpfulness	1.37	0.86	2.19	.186
Communication	1.34	0.76	2.38	.310
Interpersonal Treatment	0.92	0.43	1.97	.838
Trust	1.43	0.94	2.18	.092
Length of Time with Provider (Less than 6 months)				
Between 6 months – less than 1 year	0.47	0.31	0.72	.001
1 year to less than 3 years	1.23	1.02	1.49	.024
3 years but less than 5 years	1.08	0.98	1.19	.110
5 years or more	1.37	0.92	2.03	.112
Gender (Female)				
Male	1.00	0.84	1.19	.990
Education (Did not graduate high school)				
High school graduate or more	1.34	1.05	1.71	.016
Health Status (Fair/Poor)				
Excellent/Very Good	2.57	2.21	2.99	.000
Good	1.50	1.08	2.07	.014
Race/ethnicity (Minority)				
White, non-Hispanic	1.11	1.05	1.16	.000
Marital Status (Not married)				
Married	0.93	0.74	1.17	.559
Insurance Type (Medicaid/uninsured)				
Private	1.40	1.23	1.57	.000
Medicare	1.11	0.97	1.30	.133
Age	0.98	0.96	0.99	.002

## Discussion

Many practices have struggled with the challenge to develop fully functional patient-centered medical homes. In an effort to become more patient-centered, this study aimed to address what factors motivated diabetic patients. Understanding these factors can help identify areas of the PCMH model that practices can prioritize to emphasize patient activation. This study suggests that patients’ perceptions regarding ready access to their doctor (organizational access) and their doctor’s knowledge of their medical history (comprehensive knowledge), are important factors in motivating patients to take an active role in their health and healthcare. These findings are consistent with previous studies [25, 26] that showed modest improvement in patient activation in PCMH settings, especially in minority patients [25]; however, other similar studies show mixed results [3, 27]. Overall, there have been very few studies that has examined how the PCMH organizing model motivates patients to be more active in their healthcare.

Results of the study suggest that patients value their doctor’s comprehensive knowledge of their medical history; perhaps allowing patients to feel more confident and hence, trusting that their doctor is competent in delivering the best care based on their medical history and overall needs. Physicians/providers comprehensive knowledge of their patients’ health issues and life circumstances may lead to a more personalized care plan that meets the needs of the patient and takes into consideration their current level of self-management skills and resources available to them. Organizational access was also found to be an important factor in patient activation. Study results indicate that patients value the ability to quickly receive care and/or appointments. Similar to the aforementioned domains, office staff helpfulness and communication may also play a vital role in contributing to trust as patients are more likely to adhere to treatment when their doctor is able to give clear instructions and explanations, listens carefully and has a helpful office staff.

Unexpectedly, the results showed that interpersonal treatment was not associated with greater patient activation when we control for other PCMH characteristics. The survey question that is related to this domain asked how often their doctor spent enough time with them. Several studies have shown that patient satisfaction is linked to the patient’s perceived adequacy of the length of time they spend with their physician [28–30]. This result suggests that the time spent with the physician may not activate the patient as much as the quality of the time spent with the patient or other factors related to their office visit. The domains communication, office staff helpfulness, integration of care, interpersonal treatment, and trust became insignificant after controlling for other PCMH domain scores. This may be due to some overlapping concepts of these domains. In addition, the original ACES survey had at least 2 items for the office staff helpfulness, interpersonal treatment and trust domains and reducing these domains to a single item may impact the strength of their psychometric property.

It is important to note that this study has some limitations. First, it is a cross-sectional study, so we are unable to determine causality and the direction of the relationship between PCMH rating and patient activation. It is possible that more activated patients seek out well functioning medical practices or PCMHs. However, the family practice centers that participated in this study do not advertise themselves as PCMHs and each has a different population they serve (from inner city to suburbia) which is a strength of this study. Additionally, patients can chose their primary care doctor but there choice is usually limited by their insurance plans’ networks of participating physicians.

Our analysis did not collect data on the participants’ diabetes history, A1c levels, current treatments, or diabetes-related complications. This limitation did not allow us to determine if any of these factors may have an impact on patient activation. Another limitation of the study is that participating practices were located in a single state and were affiliated with a single academic medical center, potentially reducing generalizability. Furthermore, due to the lack of availability of electronic clinical data prior to the implementation of the electronic medical record (EMR), the authors were unable to compare pre vs. post assessment of medical home adoption. Future research should investigate, using a pre-post assessment, the impact of medical home adoption on patient activation and how improvements in each domain increases patient activation. Nevertheless, these findings have important implications regarding the adoption of PCMH concepts and practices and the need to improve organizational access and comprehensive knowledge of patients’ conditions to assist patients in having a more active role in their health and healthcare.

## Conclusions

Findings from this study contribute to an area of PCMH research that remains relatively unexplored; that is, whether or not certain attributes of medical homes are linked to levels of patient activation and ultimately better health outcomes. These findings may provide insights on the mechanism through which patients may play a more active role in their health and healthcare and hence, an increased likelihood of treatment adherence. If efforts to transform the primary care delivery system are to succeed, understanding key practice characteristics that might achieve improvements in patient activation has implications on how the PCMH model is operationalized and implemented moving forward. The development and implementation of practice strategies that improve access and providers understanding of patients’ needs can be tested and monitored using the PAM to measure improvements in patient activation. As such, findings of this study as well as future studies addressing specific strategies as noted above may inform policymakers as they advocate policies that facilitate primary care practices to transform into PCMHs.

## Abbreviations

ACES: Ambulatory Care Experiences Survey
EMR: Electronic medical record
NCQA: National Committee on Quality Assurance
PAM: Patient Activation Measure
PCMH: Patient Centered Medical Home
